# Evaluation of Immune Responses and Protective Efficacy of a Novel Live Attenuated *Salmonella* Enteritidis Vaccine Candidate in Chickens

**DOI:** 10.3390/vaccines10091405

**Published:** 2022-08-27

**Authors:** Hyunjin Shin, Tae-Min La, Hong-Jae Lee, Taesoo Kim, Seung-un Song, Eunjin Park, Gyu-Hyung Park, In-Soo Choi, Seung-Yong Park, Joong-Bok Lee, Sang-Won Lee

**Affiliations:** College of Veterinary Medicine, Konkuk University, Seoul 05029, Korea

**Keywords:** *Salmonella* Enteritidis, live attenuated vaccine, immune response, protective efficacy

## Abstract

An ideal vaccine for controlling *Salmonella* infection in chicken flocks should be safe, inducing both humoral and cellular immunity. Live attenuated vaccines against *Salmonella* Enteritidis (*S*. Enteritidis) have been used as a potential control method of *Salmonella* infection in the poultry industry. However, live attenuated vaccines can persistently infect poultry for long periods and can become virulent revertant strains. In this study, we assessed the immune responses and protective efficacy of a temperature-sensitive attenuated *S.* Enteritidis mutant as a potential vaccine candidate. In addition, we evaluated the combined vaccine administration methods to maximize both humoral and cellular immune responses in chickens induced by the vaccine candidate. Immune responses and protective efficacy were compared between the Oral/IM group, vaccinated using one oral dose at four weeks old and a booster intramuscular dose at seven weeks old, and the IM/Oral group, vaccinated using one intramuscular dose at four weeks old and a booster oral dose at seven weeks old. The Oral/IM group showed stronger immune responses than those of the IM/Oral group. Spleens from the Oral/IM group showed a promising tendency of reduction of challenged *Salmonella* compared with those of other groups. Overall, the results indicated that the *S*. Enteritidis mutant strain is a promising live attenuated vaccine candidate with good efficacy.

## 1. Introduction

A significant proportion of human *Salmonella* infections are caused by the consumption of raw or undercooked poultry products [1,2]. Notably, *Salmonella* Enteritidis (*S*. Enteritidis) is one of the most frequently reported serotypes in human *Salmonella* infections [3]. This public health concern can result in significant economic losses and can be aggravated by the antimicrobial resistances in *Salmonella* [2,4,5]. Therefore, controlling the contamination of poultry products is important for reducing salmonellosis. In addition to good hygiene and biosecurity practices, several methods have been employed to reduce *Salmonella* infections in poultry farms, such as the use of food additives, selection of chicken strains that are genetically resistant to *Salmonella* infections, and the development of *Salmonella* vaccines [6,7,8,9]. Particularly, live attenuated vaccines against *S*. Enteritidis have proven efficacy in poultry flocks [10]. Live attenuated vaccines are easy to administer and are more effective than inactivated bacterin vaccines, as they induce strong protective immunity [11,12]. However, live attenuated vaccines can persist for long periods in poultry, and high risk is associated with reversion of the vaccine to a virulent strain [13]. Therefore, the development of a safe and immunogenic strain is the biggest challenge in developing live *Salmonella* vaccines [14].

We previously developed a live attenuated *Salmonella* strain vaccine candidates using *N*-methyl-*N*′-nitro-*N*-nitrosoguanidine (not published). In this study, we evaluated its immunogenicity and protective efficacy in chickens.

## 2. Materials and Methods

### 2.1. Animals

Sixty female three-week-old specific-pathogen-free white leghorn chickens were used in this study. Upon arrival, the *Salmonella*-free status of the chickens was verified by a bacterial culture of the liver and cecum tissues of randomly selected chickens. No *Salmonella* colonies were detected in any of the tissue samples. All chickens in the experimental and control groups were maintained separately in chicken isolators. Commercial feed and drinking water were provided ad libitum. Cleaning and feeding regimes were organized to effectively prevent cross-contamination throughout the trials. The 3R principle was followed for the ethical use of experimental animals, and animal experiments were conducted under the regulations of Konkuk University’s Institutional Animal Care and Use Committee (registration number: KU21085).

### 2.2. Bacterial Strains and Culture

We used a 2S G10 strain, which is a temperature-sensitive mutant of *S*. Enteritidis developed in our laboratory. It can grow optimally at 33 °C and has a limited capability for replication at 41 °C, the body temperature of chickens. For the challenge inoculation, the 2B strain, a parental strain of 2S G10 isolated from chicken livers, was used. The 2S G10 and the 2B strains were stored as frozen cultures in tryptic soy broth (BD, Sparks, MD, USA) containing 80% glycerol at −70 °C until use. Frozen cultures were washed thrice with phosphate-buffered saline (PBS; Gibco, Paisley, UK) and adjusted to 2 × 10^7^ and 2 × 10^8^ colony-forming units (CFUs), respectively.

### 2.3. Vaccination and Challenge Inoculation

**Figure 1:**
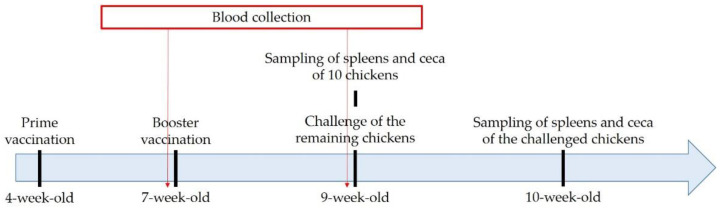
Experimental design diagram for evaluation of immune responses and protective efficacy of a vaccine candidate in chickens.

Chickens were vaccinated at four and seven weeks of age (Figure 1). Sixty chickens were equally divided into three groups (*n* = 20). The Oral/IM group of chickens was orally vaccinated at four weeks with 2 × 10^7^ CFU of the 2S G10 vaccine in 100 μL PBS and intramuscularly boosted at seven weeks with the same concentration of 2S G10. The IM/Oral group was intramuscularly vaccinated at four weeks and orally boosted at seven weeks with the same concentration of 2S G10. The control group was orally and intramuscularly inoculated with 100 μL of PBS at four and seven weeks, respectively. Ten chickens per group were euthanized two weeks post-booster vaccination, and the spleens and ceca were collected. Ten remaining chickens from each group were challenged orally with 2 × 10^8^ CFU of the 2B strain in 100 μL PBS. One week post challenge, all remaining chickens were sacrificed and the spleens and ceca were collected for the isolation of *Salmonella* strains.

### 2.4. Serological Assay

Serum was obtained after centrifugation of blood samples collected from the peripheral jugular vein of all chickens three weeks post prime and two weeks post-booster vaccination. For the detection of *S*. Enteritidis-specific antibodies, serum samples were tested using a commercially available ELISA kit (IDEXX SE Ab X2 Test kit; IDEXX Laboratories-Westbrook, ME, USA) according to the manufacturer’s instructions. The results were recorded as sample-to-positive (S/P) ratios determined by the ratio between the optical density (OD) of each sample and the mean OD of the positive control.

### 2.5. Preparation of Splenocytes

Two weeks post booster vaccination, the spleens were aseptically removed from chickens and squeezed through a 40 μm cell strainer in RPMI1640 culture medium containing 2% fetal bovine serum (FBS; Gibco, Grand Island, NY, USA) to prepare a single-cell suspension. Splenocytes were isolated by density gradient centrifugation at 400× *g* for 30 min using Histopaque-1077 (Sigma-Aldrich, St. Louis, MO, USA), washed thrice with PBS, and adjusted to 5 × 10^5^ cells/190 μL in culture medium (RPMI1640 supplemented with glutamax-I (Gibco, NY, USA), 10% FBS, 50 mM β-mercaptoethanol (Gibco, NY, USA), 100 U/mL penicillin, 100 μg/mL streptomycin, and fungizone 0.25 μg/mL (1X antibiotic–antimycotic; Gibco, NY, USA)) [15].

### 2.6. ELISpot Assay

A precoated chicken IFN-γ ELISpotPLUS kit (Mabtech, OH, USA) was used. The plate was incubated with blocking buffer (RPMI1640 medium supplemented with glutamax-I and 10% FBS) for 30 min at 22 °C. The blocking buffer was discarded and splenocytes were seeded at 5 × 10^5^ cells/well with the culture medium in triplicate. Cells were incubated in the presence of either culture medium or medium supplemented with one of the following stimulants at a final volume of 200 μL per well: concanavalin A (Invitrogen, Carlsbad, CA, USA) or soluble antigen prepared by sonicating the 2S G10 strain. The cells were incubated for 32.5 h at 37 °C and 5% CO_2_. After five washes, chicken IFN-γ (ChIFN-γ) was detected by incubation with 1 μg/mL biotinylated monoclonal antibody (MT7C10-biotin) in PBS containing 0.5% FBS (PBS-0.5% FBS) for 2 h at 22 °C. The plate was washed five times with PBS and incubated with streptavidin-HRP in PBS-0.5% FBS for 1 h at 22 °C. The plate was washed five times with PBS and ready-to-use 3,3’, 5,5’-tetramethylbenzidine substrate was added. The plate was then extensively washed in tap water, air-dried, and analyzed using an AID iSpot machine. Analyze software (Version 7.0, AID GmbH, Strassberg, Germany) allowed automated counting of the number of spots based on size and intensity.

### 2.7. Bacterial Strain Recovery

At two weeks post booster vaccination and one week post challenge, *Salmonella* strains (Log_10_ CFU/g) in the cecal contents and spleen were quantified using the plate counting method. Briefly, the cecal content was removed from the ceca. The spleens and cecal contents were weighed and homogenized. A 10-fold serial dilution was prepared to enumerate *Salmonella* strains in the processed organs. The number of bacteria was estimated by spreading the homogenate on ChromoSelect Agar (Sigma-Aldrich, MO, USA). The plates were incubated at 37 °C for 24 h.

### 2.8. Statistical Analysis

Data are presented as mean ± standard deviation (s.d.). Data were compared between the control and vaccine groups and among the vaccine groups by one-way analysis of variance followed by Tukey’s post hoc tests. Statistical analyses were performed using R (version 4.1.2, RStudio, Inc., Boston, MA, USA). Differences between the treatment groups were considered statistically significant at *p* < 0.05.

## 3. Results

### 3.1. Humoral Immune Responses Induced by Vaccination

**Figure 2:**
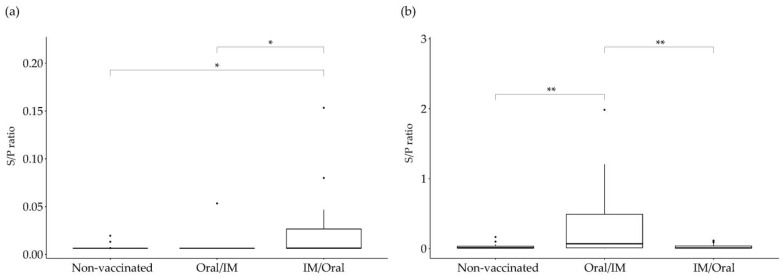
*Salmonella* Enteritidis-specific antibody titers induced by vaccination. Blood samples were collected at (**a**) three weeks after prime vaccination, and (**b**) two weeks after booster vaccination. Serum was isolated from the blood and *S*. Enteritidis-specific antibody titers were measured with a commercial ELISA kit. Each bar represents the mean sample to positive (S/P) ratio. Oral/IM, prime-boost vaccinated group with the candidate strain via oral–intramuscular route; IM/Oral, prime-boost vaccination with the candidate via intramuscular–oral route. * *p* < 0.05; ** *p* < 0.005.

The results of the serological examination after the prime and boost vaccinations are shown in Figure 2. Three weeks after the prime vaccination, significantly higher antibody titers were detected in the IM/Oral vaccine group than in the Oral/IM or non-vaccinated groups (*p* = 0.013). However, two weeks after the booster vaccination, significantly higher antibody titers were detected in the Oral/IM vaccine group than in the IM/Oral or non-vaccinated groups (*p* = 0.002).

### 3.2. Cellular Immune Responses Induced by Vaccination

**Figure 3:**
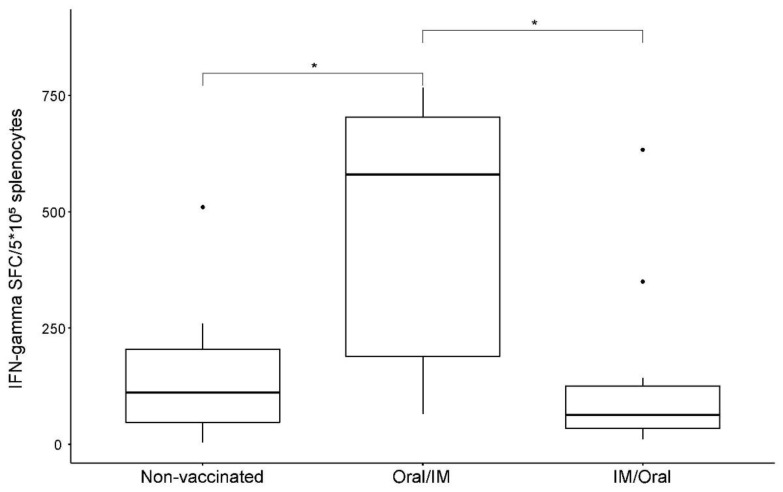
ELISpot analysis of stimulated T cells. Splenocytes collected after booster vaccination were added into chicken interferon-γ (ChIFN-γ)-coated wells and stimulated with soluble antigen for 32.5 h. The results represent the number of ChIFN-γ-secreting cells. PBS—non-vaccinated group; Oral/IM—prime-boost vaccinated group with the candidate strain via oral–intramuscular route; and IM/Oral—prime-boost vaccination with the candidate via intramuscular–oral route. * *p* < 0.05.

ChIFN-γ production by splenocytes was measured 32.5 h after stimulation with soluble antigen two weeks post booster vaccination. The results were expressed as IFN-γ spot-forming cells (SFC) per 5 × 10^5^ splenocytes. As shown in Figure 3, the number of *Salmonella* antigen-specific T cells secreting ChIFN-γ was increased in the Oral/IM group (455 ± 288 of IFN-γ SFC/5 × 10^5^ splenocytes on an average) when compared to that in the non-vaccinated group (152 ± 152 of IFN-γ SFC/5 × 10^5^ splenocytes; *p* = 0.0145. The IM/Oral group showed 143 ± 199 of IFN-γ SFC/5 × 10^5^ splenocytes on an average, which was significantly different (*p* = 0.0117) from that of the Oral/IM group.

### 3.3. Re-Isolation of Salmonella Strains

**Figure 4:**
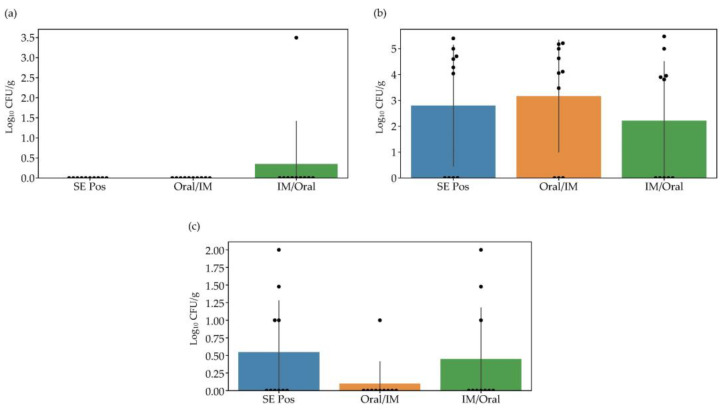
*Salmonella* titers (log_10_ CFU/g) in cecal contents before and after challenge and in the spleen after the challenge. At two weeks after vaccination and one week after challenge, ten chickens were euthanized and the *Salmonella* titers in the cecal contents and spleens were determined. (**a**) Cecal contents two weeks post booster vaccination (*p* = 0.38), (**b**) Cecal contents one week post-challenge (*p* = 0.66), and (**c**) Spleen one week post-challenge (*p* = 0.28). Spleen tissues of chickens before challenge were used for ELISpot assay. SE Pos—non-vaccinated but challenged group; Oral/IM—prime-boost vaccinated group with the candidate strain via oral–intramuscular route; and IM/Oral—prime-boost vaccination with the candidate via intramuscular–oral route.

The protective efficacy of the vaccine candidate strain against the parental strain was investigated by direct colony counting of the homogenized samples on ChromoSelect agar (Figure 4). Two weeks post booster vaccination, no *Salmonella* was detected in the cecal contents of the vaccinated chickens. Unlike the result of the prechallenge, *Salmonella* strain was found in the cecal contents after the challenge. Six, seven, and five out of ten chickens were positive for *Salmonella* in the SE Pos, Oral/IM, and IM/Oral groups, respectively. However, the number of log_10_ CFU/g was not significantly different among the groups (*p* > 0.05). Examination of spleen samples revealed that only one chicken was positive for *Salmonella* in the Oral/IM group, and the number of log_10_ CFU/g was lower than that in the SE Pos and IM/Oral groups.

## 4. Discussion

Vaccination is a valuable tool for controlling *Salmonella* infections in chickens [16,17]. A live attenuated *Salmonella* vaccine can lead to antigen presentation, resulting in high induction of specific humoral and cellular immune responses, and efficient protection against secondary infection. An ideal vaccine for the efficient control of pathogenic *Salmonella* infections should be safe and should induce both humoral and cellular immunity [18]. Commercial vaccines available against *S*. Enteritidis are mostly inactivated formulations. These vaccines mainly stimulate antibody production and only have antigens present at the time of in vitro harvesting. Therefore, most of the current *S*. Enteritidis vaccines induce insufficient immune response to protect chickens from *Salmonella* infection [19,20]. In this study, we evaluated the immunogenic potential and protective efficacy of a novel formulation of *S*. Enteritidis strain as a vaccine candidate using a combination of two different inoculation routes. Oral inoculation is economical, easy to apply and one of the most commonly recommended methods for mass vaccination in the poultry industry [21,22]. Other researchers have shown that oral vaccination with a *S*. Enteritidis live vaccine reduced shedding of the challenged strain after challenge infection and induced significantly better response of splenocytes against *S*. Enteritidis-flagella compared to that of the unvaccinated chickens [7,23]. The efficacy test of *S*. Gallinarum 9R vaccine strain evaluated using 10-week-old white leghorn chickens showed partial protection after oral and subcutaneous vaccination as three and one out of twenty liver cultures were found to be positive, respectively [24]. These results indicated that oral administration of a *Salmonella* live vaccine could not provide sufficient protection to the chickens against the systemic *Salmonella* infection compared to subcutaneous administration. Intramuscular inoculation requires more labor, but has been related to higher antibody responses [16,25]. Previous data demonstrated that intramuscular inoculation of a live *S.* Gallinarum vaccine elicited clear humoral immune responses in which immunoglobulin G concentration was significantly higher than the PBS control group 1 week post-vaccination [26]. Moreover, there is a result that chickens received two intramuscular injections of *S*. Gallinarum vaccine at 10 and 14 weeks old, which showed the strongest and more long-lasting antibody responses than the group receiving two oral inoculation or a single injection [27]. These previous results indicated that oral inoculation of *Salmonella* live vaccines can induce a high cellular immune response, while subcutaneous or intramuscular administration can induce a high humoral immune response. Both immune responses are required to sufficiently protect birds from local and systemic *Salmonella* infection. Therefore, we evaluated oral and intramuscular combined vaccine administration methods and compared prime-boost vaccination with the vaccine candidate via oral–intramuscular and intramuscular–oral routes, respectively, to strengthen the advantages of each inoculation route.

Cellular immunity protects animals against intracellular pathogens by activating cytotoxic and helper T-lymphocytes, macrophages, and natural killer cells [28,29]. These activated T-lymphocytes can produce Th1 and Th2 immune responses mediated by cytokines or by directly destroying foreign organisms [28]. In this study, we investigated the Th1 response by examining the number of *Salmonella*-specific T-cells that produce ChIFN-γ, the most important cytokine during the early phase of infection by intracellular pathogens [28]. The chickens in the Oral/IM group showed significantly higher levels of IFN-γ cytokine responses than those in the non-vaccinated group, whereas no significant difference was noticed between the IM/Oral and non-vaccinated groups. This suggested that primary vaccination through the oral route and intramuscular booster mainly induced Th1 immune response. Th2 cells produce several cytokines that are important for the induction of humoral immune responses and antibody production [30]. Monitoring serum antibodies after each vaccination revealed that the prime-boost immunizations using different routes induced a significant increase in the S/P ratio. Intramuscular priming induced a significantly higher IgG response than oral priming three weeks after vaccination. However, the chickens in the Oral/IM group, which received prime vaccination via the oral route, showed a significant increase in IgG levels when administered with a booster dose via the intramuscular route. On the other hand, no significant differences in IgG levels were noticed in the IM/Oral group after oral boosting. These results indicated that the intramuscular administration of live attenuated *S*. Enteritidis induces a strong humoral immune response, regardless of the order of prime-boost vaccination.

The prime dose of vaccination induces adaptive immune responses through the activation of B or T lymphocytes, which either leads to antibody production or cellular immunity, respectively, and subsequent exposure to a booster dose further enhances the immune responses via memory cells formed after prime vaccination [25]. Although oral vaccination did not increase antibody titers, their increase after intramuscular booster vaccination suggests that oral vaccination also can generate memory B cells to some extent.

Protection efficacy appeared to be partially dependent on the prime-boost strategy. Although no significant differences were observed among the groups regarding protective efficacy at one week after challenge, a decreased log_10_ CFU was noticed for the spleens in the Oral/IM group. Moreover, we assumed that re-isolated *Salmonella* are not the 2S-G10 strain, since there is a datum indicating that the 2S-G10 strain disappears in a week when inoculated to 1-day-old chicks (not published). Therefore, prime and booster vaccinations via the oral and intramuscular routes could induce protective effects in chickens, as these findings also correlated with the data of immune response; therefore, increasing the cellular immune responses through oral vaccination is necessary to improve protection against *Salmonella* infection.

## 5. Conclusions

The results of this study indicate that our live attenuated *S*. Enteritidis strain can be used as a vaccine with an optimized immunization strategy via the oral–intramuscular route to prevent *Salmonella* infection in chickens and contribute significantly to establishing consumer confidence in safe poultry products.

## Data Availability

Not applicable.
